# Pan-American Medical Congress Executive Committee

**Published:** 1892-02

**Authors:** Charles A. L. Reed

**Affiliations:** Secretary General; Cincinnati


### The International Executive Committee of the Pan-American Medical Congress.

The Committee on Organization of the Pan-American Medical Congress at its meeting at St. Louis last October, elected the following International Executive Committee: The Argentine Republic, Dr. Pedro Lagleyze, Beunos Ayres; Bolivia, Dr. Emelio Di Tomassi, La Paz; Brazil, Dr. Carlos Costa, Rio de Janeiro; British North America, Dr. Jas. F. W. Ross, Toronto; British West Indies, Dr. James A. DeWolf, Port of Spain; Chili, Dr. Moises Amaral, Santiago; United States of Columbia, Dr. P. M. Ibañez, Bogota; Costa Rica, Dr. Daniel Nuñez, San José; Ecuador, Dr. Ricardo Cucalon, Guayquil; Guatemala, Dr. José Monteris, Guatemala Nueva; Hayti, Dr. D. Lamothe, Port au Prince; Spanish Honduras, Dr. George Bernhardt, Teguagalpo;

Mexico, Dr. Tomás Noriega, City of Mexico; Nicaragua, Dr. J. I. Urtecho, Grenada; Peru, Dr. J. Casamira Ulloa, Lima; Salvador, Dr. David J. Guzman, San Salvador; Spanish West Indies, Dr. Juan Santos Fernandez, Habana; United States, Dr. A. Vander Veer, Albany, N. Y.; Uruguay, Dr. Jacinto De Leon, Montevideo; Venezuela, Dr. Elias Roderiguez, Caracas.

Hawaii, Paraguay, Santo Domingo, the Danish, Dutch and French West Indies are not yet organized. Nominations of local officers have been received from a majority of all the members of the International Executive Committee, and a number of the lists have been confirmed by the Committee on Organization. These will be announced as rapidly as acceptances are received.

CHARLES A. L. REED,

Secretary General.

Cincinnati, January 15, 1892.

